# Using generative adversarial networks for genome variant calling from low depth ONT sequencing data

**DOI:** 10.1038/s41598-022-12346-7

**Published:** 2022-05-30

**Authors:** Han Yang, Fei Gu, Lei Zhang, Xian-Sheng Hua

**Affiliations:** 1 City Brain Lab, DAMO Academy, Alibaba Group, Hangzhou, China; 2 Department of Computing, The Hong Kong Polytechnic University, Hung Hom, Hong Kong

**Keywords:** Genetics research, Computational models, Machine learning

## Abstract

Genome variant calling is a challenging yet critical task for subsequent studies. Existing methods almost rely on high depth DNA sequencing data. Performance on low depth data drops a lot. Using public Oxford Nanopore (ONT) data of human being from the Genome in a Bottle (GIAB) Consortium, we trained a generative adversarial network for low depth variant calling. Our method, noted as LDV-Caller, can project high depth sequencing information from low depth data. It achieves 94.25% F1 score on low depth data, while the F1 score of the state-of-the-art method on two times higher depth data is 94.49%. By doing so, the price of genome-wide sequencing examination can reduce deeply. In addition, we validated the trained LDV-Caller model on 157 public Severe acute respiratory syndrome coronavirus 2 (SARS-CoV-2) samples. The mean sequencing depth of these samples is 2982. The LDV-Caller yields 92.77% F1 score using only 22x sequencing depth, which demonstrates our method has potential to analyze different species with only low depth sequencing data.

## Introduction

Genome variant plays a vital role in many fields. For human beings, recent works^1–3^ present us that knowing the entire DNA sequence of the diploid human and studying genome variants can facilitate the understanding of cancer and contribute substantially to deciphering genetic determinants of common and rare diseases. Moreover, genome variant determines evolutions among virus strains such as the latest coronaviruses^4^. In addition, single nucleotide polymorphism (SNPs) variant is the fundamental part for animal and plant breeding^5^. Therefore, the research about genome variant calling is urgently to have deep improvement.

To obtain individual genome, two kinds of genome-wide sequencing technologies, next generation sequencing (NGS) and third generation sequencing (TGS), have been developed in recent years based on distinct sequencing mechanism^6–10^. In NGS, we use Illumina sequencer as example, single DNA is sonicated into pieces of fragments (known as reads) with length between 50-300 base pair (bp, length unit of DNA sequence), and each base of the massive reads is then detected by sequencer based on the fluorophores color in parallel. On the other hand, TGS (e.g., Oxford Nanopore Technology (ONT)) used the single molecule sequencing (SMS) technology to detect the entire DNA molecule in real time, with length more than 10 thousand bp. TGS has potential for structural variant analysis but higher error rate, which is 0.1-1% in NGS and up to 15% in TGS. In this work, we focus on ONT data from TGS.

**Figure 1:**
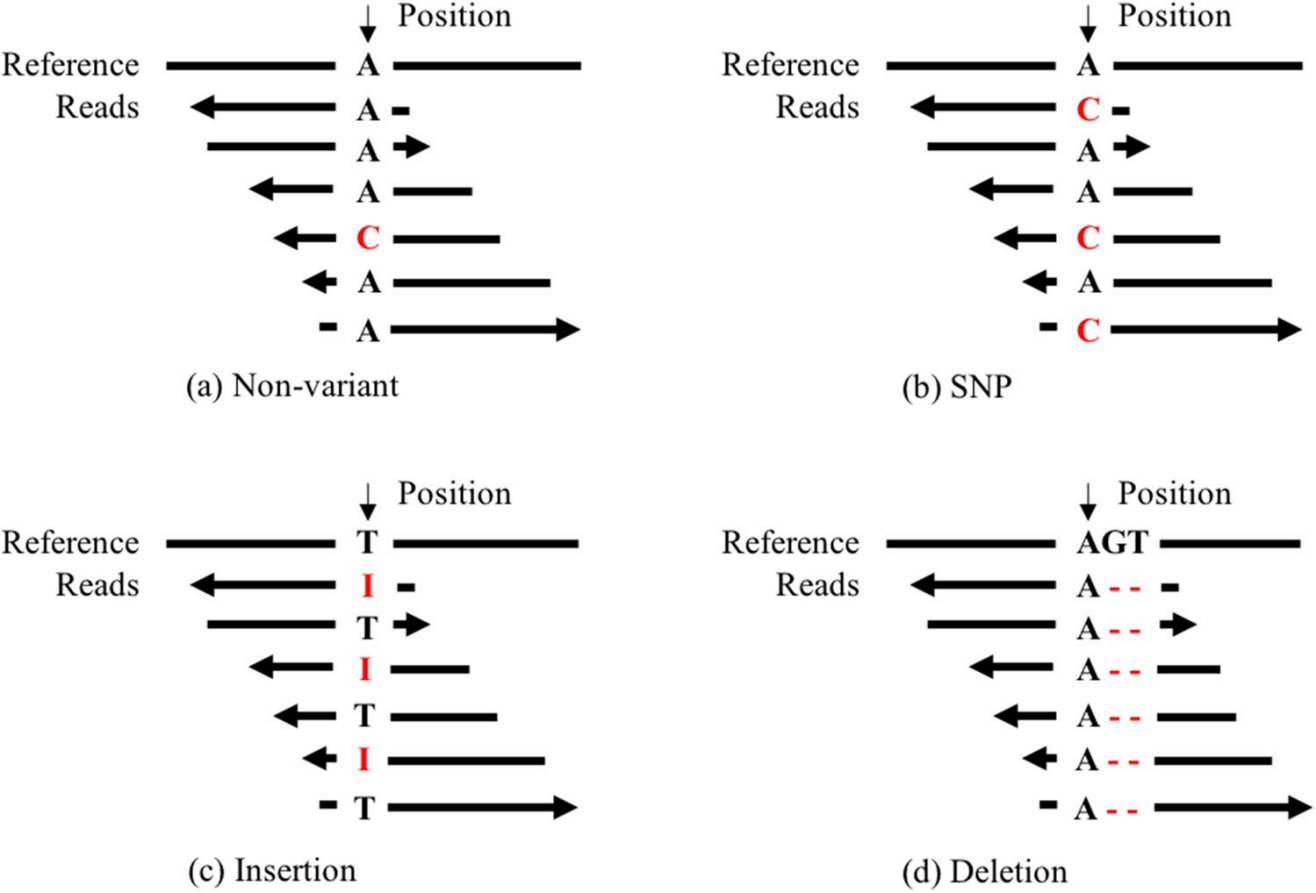
Examples of alignments for different genome variants. For easy understanding, we simplify illustration of the alignment. Each alignment consists of reference sequence and related reads. (**a**) is extracted at a non-variant genome site, where a difference C is from sequencing error. (**b**) indicates a heterozygous SNP variant with allele C. (**c**) is a heterozygous Insertion variant, for instance, from T to TAAT. (**d**) is a homozygous Deletion variant, i.e., from AGT to A. (**c**) and (**d**) are known as Indels variant.

**Figure 2:**
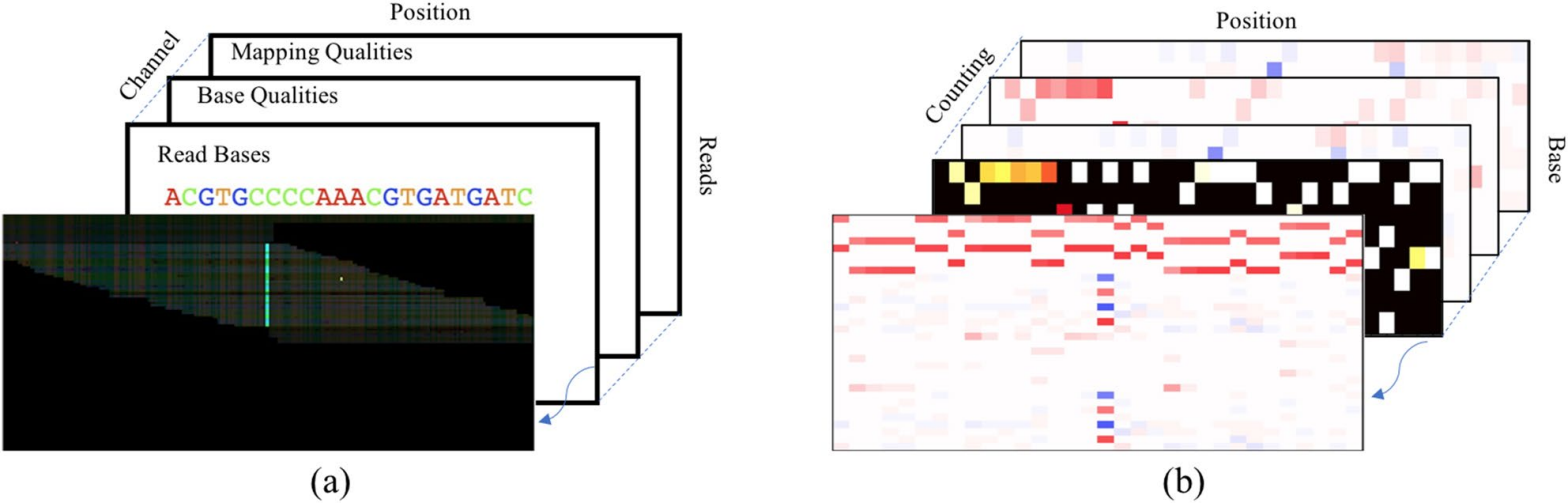
Illustration of input pileup images for two typical methods. (**a**) is for the DeepVariant, and (b) is for the Clair. The front images of (a) and (b) are reconstructed from the rest slices. (a) is a color image of size $200\times 100$ with multiple channels, e.g., read base, base quality, mapping quality, etc. (**b**) is a three-dimension image of size $33\times 8\times 4$ containing four kinds of counting information.

There are two major types of variations: (1) single nucleotide polymorphism (SNPs), with only one positions of mutation as shown in Fig. 1b, and (2) insertion or deletion (Indels) in Fig. 1c–d, with unlimited bases of insertions or deletions compared with reference genome. For diploid species like human being, there are three genotypes (GT): no variations in each allele (homozygous reference), variation in one allele but not in the other (heterozygous), and variations in both alleles (homozygous mutation). Variation calling is a computational driven task due to the existence of large amounts of variant sites in a genome. For example, in human genome, there are millions of known short variation sites in 3 billion genome^11^. Genome variant calling actually aims to (1) find the variant site, i.e., distinguish variants in Fig. 1b–d from large amounts of non-variant candidates shown as Fig. 1a, (2) know the mutations (i.e., alternative alleles), (3) clear whether two DNA chains all have the mutation (i.e., Genotype).

As discussed above, genome variant calling is critical but quite challenging due to the large amounts of variant sites and error-prone of the sequencing technology. Especially for the TGS, it has much higher frequency of variant candidates and up to 15% error rate. Recent works can be divided into two categories, traditional methods^11–14^ and deep learning based methods^15–18^. The traditional methods usually need manual interaction and hand-crafted features, which limits its performance. Moreover, it is notable that most traditional methods have poor operation efficiency. For example, Medaka caller^14^ even needs 7.5 days to process a single ONT sample of human. Since Deepvariant caller^17^ transformed genome variant calling to image classification task shown as Fig. 2a, deep learning-based methods have become the baseline in this field. The Clair caller^16^, the successor to Clairvoyante^15^ is the state-of-the-art (SOTA) approach for germline variant calling on the public ONT data. However, these methods almost rely on high depth sequencing data which is expensive to obtain. We found that performance of the SOTA method Clair for ONT data drops significantly on low depth data. Moreover, the price of genome-wide sequencing examination is positively correlated with its coverage rate. If genome variant calling can be yielded from low depth sequencing data, the corresponding cost can reduce deeply.

In this work, in order to address these issues, we developed a low depth variant calling method (LDV-Caller) based on the SOTA Clair model. The LDV-Caller utilized generative adversarial network to aid the Clair model processing low depth sequencing data. In addition, we validated the proposed method on two public genome datasets, i.e., human genome data from GIAB^19^ and SARS-CoV-2 data from NCBI^20^. In a summary, our main contributions are as follows:

- We propose the LDV-Caller, a low depth variant caller, which can project low depth data to higher one via using an encoder-decoder generative model.
- To obtain more complete sequencing information, we introduce adversarial learning to facilitate training.
- All components in the LDV-Caller can be trained end-to-end. Experimental results on low depth ONT data show that our approach outperforms SOTA method.

## Results

### Results on 0.5 ds low depth data

First, we examined whether the proposed LDV-Caller does have improvement on low depth data. In this work, we used dataset from Genome in A Bottle (GIAB) consortium^19,21^ because of the integration of multiple variant calling program on a bunch of datasets, and the special process steps on variant sites among the programs. It provided all kinds of necessary data for genome analysis, e.g., reference human genome sequence (FASTA file), binary alignment map (BAM file) built from sequenced reads, high confidence regions (BED file), truth genome variants (VCF file), etc. We used three samples, i.e., HG001, HG002, and HG003 from Oxford Nanopore (ONT) machine. And BAM files of HG001, HG002 and HG003 are down-sampled to have half sequencing depth (noted as 0.5ds, where ’ds’ represents down-sampling rate). For the above purpose, sample HG001 is first used to train the models while the whole different sample HG002 is for testing. The trained LDV-Caller and Clair models are compared on 0.5ds down-sampled low depth data of HG002. Table 1 reports detailed results. From the 1st and 3rd rows of Table 1, we can find that overall F1 score of Clair method drops 2.7% from 94.49% to 91.79%, when HG002 has half the original sequencing depth. It’s also notable that there are millions of variant sites in singe human genome. So, hundreds of thousands of variants are not detected, which is unacceptable. The 4th row of Table 1 presents that our LDV-Caller can develop the Clair method to process well on 0.5ds down-sampled low depth data. Overall F1 score on 0.5ds low depth data increases to 94.25% which is very close to the result of the Clair method on two times higher depth data. Since there are multiple submodules in the LDV-Caller model. We also designed the ablation study experiments on 0.5ds data. In Table 2, the 1st and 3rd rows are actually the Clair and the LDV-Caller method. From Table 2, we can see that both generative model and adversarial discriminator are important.

**Table 1:** Experimental results on GIAB dataset. We used chromosomes 1 to 22 of samples HG001, HG002, and HG003. The suffix ’ds’ indicates down-sampled rate. Both SNPs and Indels are used to make overall evaluations.

Method	DS rate	Train data	Test data	Precision	Recall	F1 score	TP	FP	FN
Clair	1.0ds	HG001:chr1-chr22	HG002:chr1-chr22	0.9524	0.9376	0.9449	2,815,372	140,722	187,362
Clair	1.0ds	HG001:chr1-chr22	HG003:chr1-chr22	0.9555	0.9331	0.9441	2,675,506	124,673	191,895
Clair	0.5ds	HG001:chr1-chr22	HG002:chr1-chr22	0.9344	0.9020	0.9179	2,708,574	190,127	294,101
LDV-Caller	0.5ds	HG001:chr1-chr22	HG002:chr1-chr22	0.9622	0.9235	**0**.**9425**	2,773,166	108,865	229,584
Clair	0.5ds	HG002:chr2-chr22	HG002:chr1	0.9323	0.8999	0.9158	214,797	15,588	23,887
LDV-Caller	0.5ds	HG002:chr2-chr22	HG002:chr1	0.9581	0.9200	**0**.**9386**	219,592	9,615	19,095
Clair	0.3ds	HG001:chr1-chr22	HG002:chr1-chr22	0.8289	0.7716	0.7992	2,316,904	478,278	685,727
LDV-Caller	0.3ds	HG001:chr1-chr22	HG002:chr1-chr22	0.9300	0.8601	**0**.**8937**	2,582,572	194,462	420,102
Clair	0.5ds	HG001:chr1-chr22	HG003:chr1-chr22	0.8924	0.8479	0.8705	2,436,327	293,749	431,043
LDV-Caller	0.5ds	HG001:chr1-chr22	HG003:chr1-chr22	0.9476	0.9102	**0**.**9285**	2,609,887	145,095	257,491

**Table 2:** Experimental results for ablation study on 0.5ds data. HG001 is the training data while HG002 is for testing. Since the LDV-Caller has extra submodules *G* and *D* than the Clair *C*, we made two experiments to validate the effectiveness of both *G* and *D*. The $\checkmark$ indicates whether the certain submodule is used. The first row is actually the Clair method while the last row is our LDV-Caller. MSE is used to evaluate submodule *G* while other metrics are used to evaluate the whole LDV-Caller.

Modules			Metrics			
*C*	*G*	*D*	MSE	Precision	Recall	F1 Score
$\checkmark$			-	0.9344	0.9020	0.9179
$\checkmark$	$\checkmark$		0.1996	0.9516	0.9127	0.9317
$\checkmark$	$\checkmark$	$\checkmark$	0.2014	0.9622	0.9235	**0**.**9425**

Significant value in bold.

To eliminate the specificity of testing data, we also test the trained LDV-Caller and Clair models on another sample (HG003). From the 2nd and 9th rows of Table 1, the overall F1 score drops 7.36% from 94.41% to 87.05% on 0.5ds low depth data. This is much worse than result on HG002. Fortunately, our LDV-Caller achieves 92.85% overall F1 score as shown in the 10th row of Table 1, which is much better than the Clair method. To test whether the proposed method might have any tendency to produce genome position-specific error, we trained the model on chromosomes 2 to 22 of HG002 while the rest chromosome 1 was used as test data. There are 248.9 million sites in chromosome 1 which is nearly one-tenth of the whole genome. From the 5th and 6th rows of Table 1, there is also 2.28% improvement of the overall F1 score. Based on the above results, we can find that the proposed LDV-Caller method does process well on 0.5ds low depth data.

### Results on 0.3 ds low depth data

**Figure 3:**
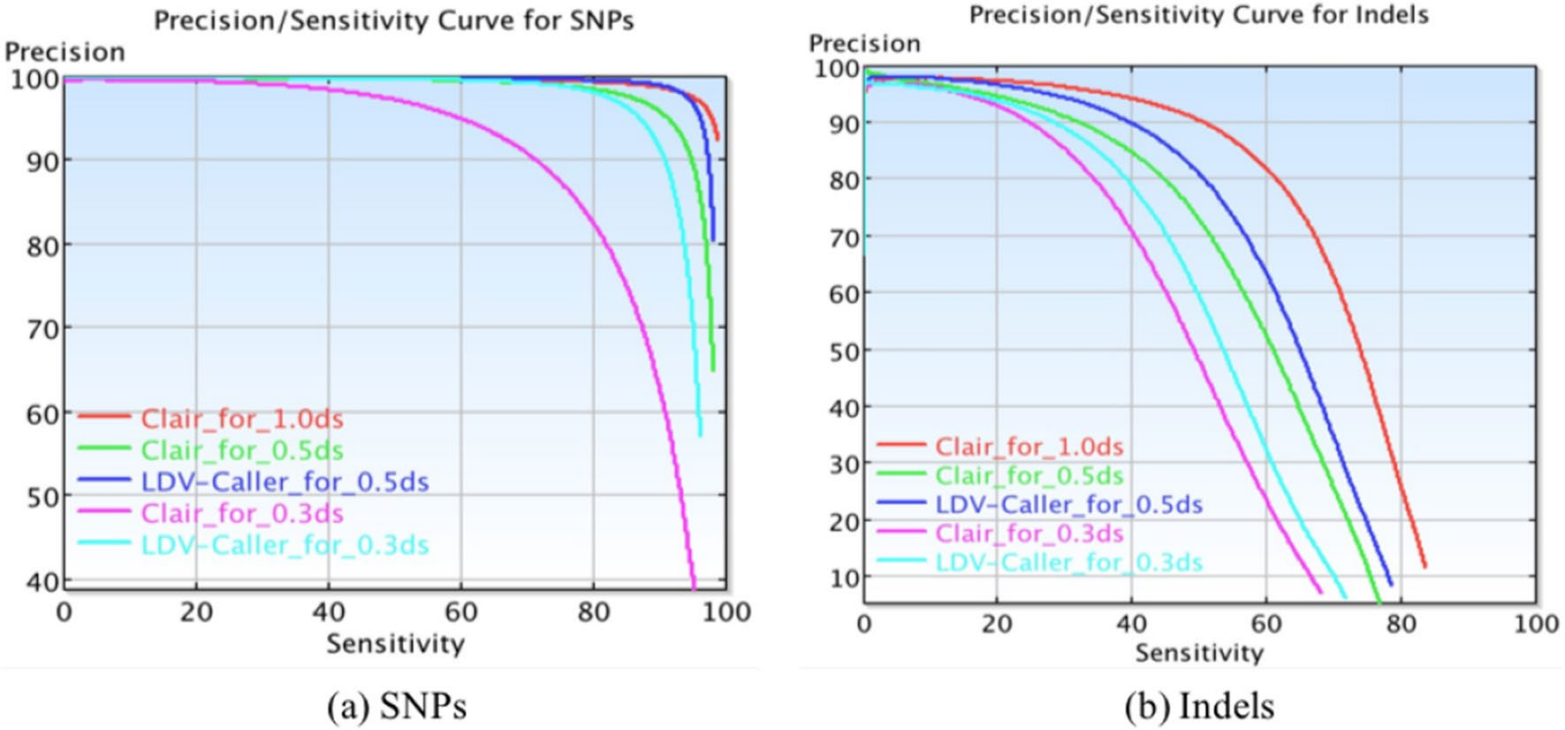
Precision/Sensitivity curves for SNPs and Indels on HG002.

**Figure 4:**
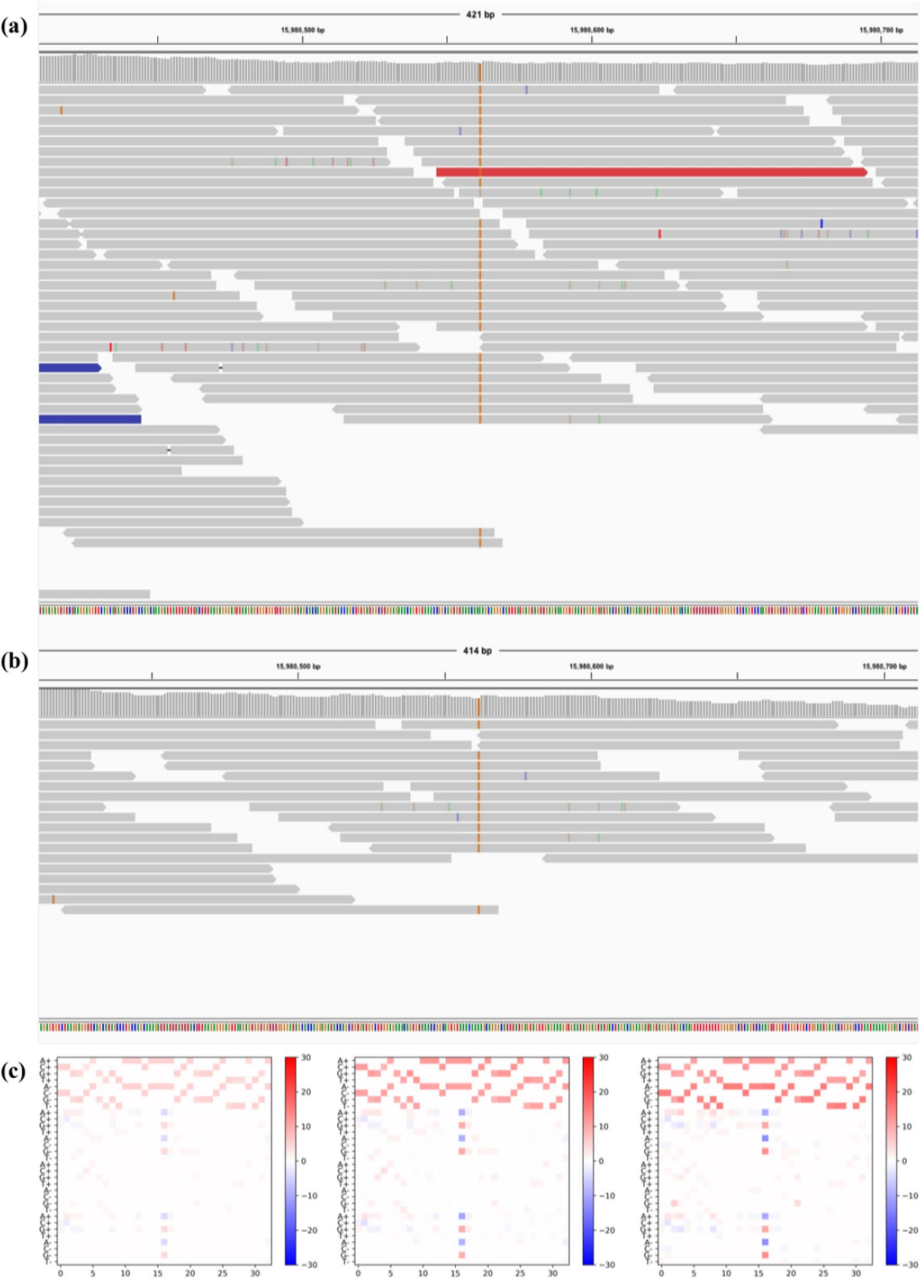
Example of IGV visualization. The variant is at chr21:15,980,562 of HG002. This variant is not detected by Clair method on 0.3ds down-sampled data. But our LDV-Caller correctly found it from 0.3ds data and gave a high confidence. (**a**) is the alignment of original high depth data. (**b**) is the alignment of 0.3ds down-sampled data. (**c**) contains three pileup images. From left to right, they are low depth pileup image, predicted “high” depth pileup image, and real high depth pileup image respectively.

In order to make further validation of the proposed method, we even down-sampled the original BAMs to have 0.3 times of their sequencing depth (noted as 0.3ds). Then the Clair method and our LDV-Caller are used to analyze 0.3ds down-sampled BAMs. As reported in Table 1, overall F1 score on 0.3ds low depth data of HG002 decreases 14.57% from 94.49% to 79.92%, which is much worse than the result on 0.5ds low depth data. This indicates the Clair method is very sensitive to the depth of the sequencing data. However, high depth sequencing data is not always provided in clinical practice. Fortunately, our LDV-Caller achieves 89.37% overall F1 score on 0.3ds low depth data. There is around 10% improvement than the Clair method on this extreme low depth data. Furthermore, Figure 3 presents us Precision/Sensitivity curves for both SNPs and Indels on HG002. The proposed method has better performance on SNPs than Indels. For more intuitive understanding, we also made analysis of the trained LDV-Caller model. As shown in Fig. 4, the variant at chr21:15,980,562 of HG002 is not detected by Clair model on 0.3ds data. But our LDV-Caller model found it and gave a high confidence. We also visualized related pileup images in Fig. 4c, i.e., pileup image extracted from 0.3ds data, predicted pileup image by the LDV-Caller model, and pileup image extracted from original data. We can find that the LDV-Caller projected high depth sequencing information from low depth data.

### Results on SARS-CoV-2 data

Since December 2019, COVID-19 disease is still spreading all over the world. Severe acute respiratory syndrome coronavirus 2 (SARS-CoV-2) is the causative pathogen for COVID-19 disease. Analyzing SARS-CoV-2 can help curing COVID-19 disease. Therefore, we also examined whether the proposed method can be used to call variants from low depth SARS-CoV-2 data. We used 157 public SARS-CoV-2 samples from NCBI website^20^. The mean sequencing depth of these samples is 2982. We used result of the Medaka method as ground truth because of lacking truth variants for existing public genome data. We first down-sampled all SARS-CoV-2 samples to have 22x sequencing depth. The trained LDV-Caller was then used to make variant calling on all down-sampled data. Our LDV-Caller yields 92.77% F1 score using only 22x sequencing depth data as reported in Table 3, which demonstrates our method can be used to analyze different species with low depth sequencing data.

**Table 3:** Experimental results on SARS-CoV-2 dataset.

Method	Depth	Precision	Recall	F1 score
Clair	22x	0.9170	0.9123	0.9146
LDV-Caller	22x	0.9392	0.9165	**0**.**9277**

Significant value in bold.

## Discussion

As discussed in the last section, we can obtain the following key points: (1) The Clair, the SOTA method on ONT data, is sensitive to the depth of sequencing data. (2) Our method, LDV-Caller, can use 0.5ds low depth data to yielding close results performed by the Clair model on two times higher depth data. By doing so, the price of genome-wide sequencing examination can reduce deeply. This will further encourage the wide application of genome analysis. (3) Our LDV-Caller is more suitable for SNP variant calling from low depth data. More than 95% variants are SNPs in human genome. Moreover, SNP variants are the fundamental part for animal and plant breeding. Therefore, the proposed method has great value in practice. (4) Experimental results on SARS-CoV-2 data not only validate the effectiveness of the proposed method, but also indicate that this method has potential to process different species.

As far as we know, we are the first to make research on genome variant calling from low depth sequencing data, which is significantly valuable in genome analysis procedure. In this work, we mainly focus on TGS data, so we use Clair model as variant caller. For NGS data, Deepvariant yielded SOTA result. If performance of Deepvariant also relies on sequencing depth, our method can be easily used to incorporate with Deepvariant model. The proposed LDV-Caller moves the research of genome variant calling from low depth sequencing data a step forward. Although the result of genome variant calling on low depth ONT data has been largely improved, there are still some weakness to solve and some potential works to try. For instance, the performance on Indels still need to have improvement. In current aggregated statistical images, details of sequencing reads are neutralized, it is recommended to use original reads information to generate images. In addition, it’s worth noting that all GIAB samples used in this work were generated by the same chemistry kits, i.e., SQK-LSK109 library prep kits on the FLO-PRO002 flow cell, which is a possible limitation factor. We will make further validation in the future work.

## Methods

### Overview

**Figure 5:**
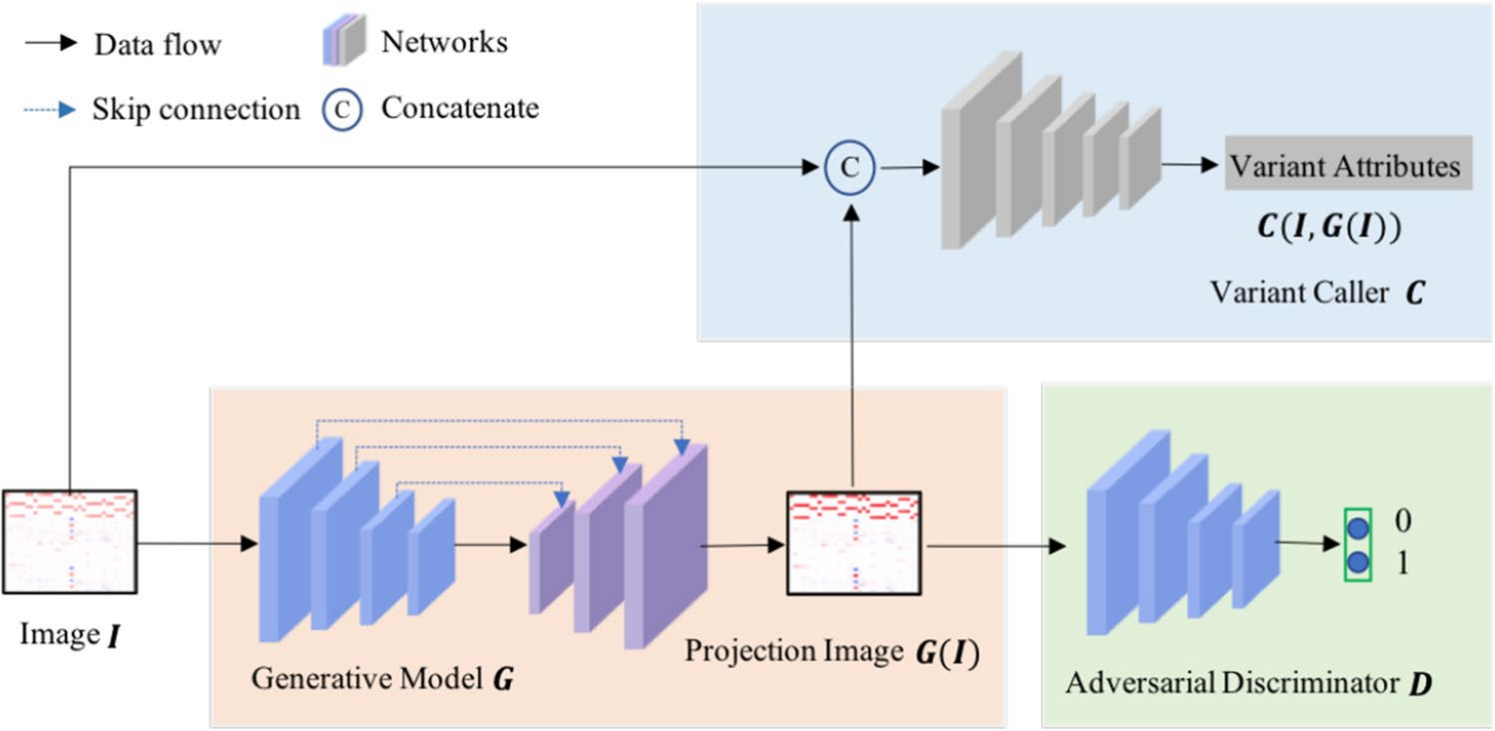
The framework of our LDV-Caller. Three sub-models, generative model *G*, adversarial discriminator *D*, and variant caller *C* are included. Detailed structures are in Table 4.

As illustrated in Fig. 5, the LDV-Caller consists of three sub-models, i.e., generative model *G*, variant caller *C*, and adversarial discriminator *D*. Image *I* and $I_{gt}$ are a pair of training sample extracted from low depth and high depth data. *G* is first used to generate the projection image *G*(*I*). Then *C* takes concatenation of *G*(*I*) and *I* as input and predicts four types of variant attributes to make final decision as^16^. *D* is a binary classifier which can provide adversarial learning to facilitate optimizing the LDV-Caller. It’s notable that *D* is no longer needed at testing time.

Table 4 reports the detailed structures of our LDV-Caller. In the following, we will delve into each submodule and loss function to clarify the entire method.

**Table 4:** The detailed layers in our LDV-Caller. k, d, c respectively represent kernel size, dilation radius, channel size.

Module	Details
*I*	counting based pileup features, $33\times 8\times 4$
	convert to 2D image, $33\times 32\times 1$
*G*	(conv2d, conv2d, maxpool2d), (k=3, d=1,2, c=16)
	(conv2d, conv2d, maxpool2d), (k=3, d=1,2, c=32)
	(conv2d, conv2d, maxpool2d), (k=3, d=1,2, c=64)
	(conv2d, conv2d), (k=3, d=1,2, c=64)
	(deconv2d, concat, conv2d, conv2d), (k=3, d=1,2, c=64)
	(deconv2d, concat, conv2d, conv2d), (k=3, d=1,2, c=32)
	(deconv2d, concat, conv2d, conv2d), (k=3, d=1,2, c=16)
*D*	backbone has same structure with encoder of *G*
	global-avgpooling layer, hidden size=64
	fully-connected layer with sigmoid, hidden size=1
*C*	BiLSTM, layer=2, hidden size=128
	2 fully-connected layers, hidden size=192, 96
	4 output fully-connected layers with softmax, hidden size=# of classes

### The LDV-Caller

#### Pileup image features

There are two methods for generating input features from the binary aligned map (BAM) data for deep learning models. One is proposed in DeepVariant^17^. As shown in Fig. 2a, they pile up all reads covered the candidate variation position into a 2D color picture. Different types of feature are included in different channel. The features include read base, strand of the read, base quality, and mapping quality, etc. Although this method includes almost all useful information, the input size is too huge to restrict its processing speed^15^ and^16^ leverage another counting strategy to generate the input features like Fig. 2b. The size of the input is respectively $33\times 4\times 4$ and $33\times 8\times 4$, which is more convenient to process. In this paper, we use the same input features as^16^, which added useful strand information compared with^15^.

Figure 2b shows the input features of our LDV-Caller model. It has three dimension, i.e., base position, read base and counting strategy. The position dimension centers at the candidate position*c* and ranges in $[c-a, c+a]$, where *a* is the flanking window. The read base dimension consists of A, C, G, T, A-, C-, G- and T-, where A, C, G, T are from forward strand while A-, C-, G-, T- are from reverse strand. The third dimension includes various statistic ways. In this paper, we use (1) the allelic count of the reference allele, (2) insertions, (3) deletions, and (4) single nucleotide alternative alleles. These three-dimension tensors with size of $33\times 8\times 4$ compose our input for the LDV-Caller.

#### Variant caller

In this paper, we choose model *C* proposed in^16^ as the variant attribute extractor for two reasons, i.e., effectiveness and efficiency. At first^16^, achieves the state-of-the-art results for germline variant calling on Oxford Nanopore Technology (ONT) data. At the same time, it is at least 7 times faster than both traditional method Longshot^11^ on ONT data and deep learning based method DeepVariant^17^ on NGS data.

The model *C* has four types of variant attributes to predict. They are: (1) genotype with 21 classes, (2) zygosity with 3 classes, (3) length of allele 1 with 33 classes, and (4) length of allele 2 with 33 classes. Based on these predictions^16^, also proposed an algorithm to determine the most probable genotype and corresponding alternative alleles. We use focal loss^22^ as our loss function, and parameter $\gamma$ is set to 2. Furthermore, we treat each task equally. Therefore, model *C* is supervised by $\mathscr {L}_C$, as:

1 $$\begin{aligned} {\mathscr {L}_C} = {\mathscr {L}_{21}} + {\mathscr {L}_{3}} + {\mathscr {L}_{33}} + {\mathscr {L}_{33}^{'}}, \end{aligned}$$

where, $\mathscr {L}_{21}$, $\mathscr {L}_{3}$, $\mathscr {L}_{33}$, $\mathscr {L}_{33}^{'}$ are respectively losses for four variant attribute prediction tasks.

#### Generative model

 Similar to image quality enhancement^23^, the generative model *G* is proposed to build relation between two data distribution domains, i.e., from low depth domain to high depth domain. It’s an encoder-decoder network of which input and output have the same size. We also introduce skip connections and dilated convolutional layers into model *G*. Table 4 shows the detailed layers. For each variant candidate, we first respectively extract 3D pileup images with size of $33\times 8\times 4$ from low depth and corresponding high depth data. Whereas, the last two dimensions of these 3D pileup images, especially the third dimension, have too small size to make model *G* very deep. Deep model with more pooling operations is the key to extract high level semantic features. For these reasons, 3D pileup images are then flattened along the third dimension to get 2D images *I* and $I_{gt}$ with size of $33\times 32$. As shown in Fig. 5, *G* takes image *I* as input and generates a predicted high depth image, i.e., the projection image *G*(*I*). We use mean square error as the loss function to optimize model *G*. It is calculated as follow:

2 $$\begin{aligned} {\mathscr {L}_{G}} = {\parallel {G(I) - I_{gt}}\parallel }_2 \end{aligned}$$

#### Adversarial discriminator

In order to recover more complete information from low depth data, we also introduce adversarial learning into our method, which has proven its effectiveness for better image transformation. Specifically, the adversarial discriminator *D* is a binary classifier. Details are given in Table 4. The adversarial discriminator *D* and the generative model *G* are like playing a minimax two-player game^24^. Model *D* aims at correctly distinguishing predicted high depth image from truth high depth image, while the generative model *G* wants to generate enough realistic high depth image to confuse model *D*. Therefore, adversarial learning has been used to promote model *G*. For each training step, model *D* is inferred by two times. At first, when training model *G*, it provides extra adversarial loss $\mathscr {L}_{adver}$, as:

3 $$\begin{aligned} {\mathscr {L}}_{adver} = -{\mathbb {E}}[\log (D(G(I),I))]. \end{aligned}$$

Then, when training model *D*, corresponding loss is $\mathscr {L}_{D}$, as:

4 $$\begin{aligned} {\mathscr {L}}_{D} = -{\mathbb {E}}[\log {D(I_{gt}}, I) + \log (1-D(G(I), I))] \end{aligned}$$

### Loss function

All components in the LDV-Caller are trained end-to-end. However, due to the existing of adversarial learning, there are two optimizers during training. For each iteration, parameters of model *G*, *C* are first updated at the same time. They are supervised by loss $\mathscr {L}$, as:

5 $$\begin{aligned} \mathscr {L} = \lambda _1\mathscr {L}_{G} + \lambda _2\mathscr {L}_{C} + \lambda _3\mathscr {L}_{adver}, \end{aligned}$$

where $\lambda _1, \lambda _2$ are set to 1, while $\lambda _3$ is set to 0.1 in this work. After that, the model *D* is supervised by loss $\mathscr {L}_{D}$.

### Implementation details

#### Datasets

In this work, we used dataset from Genome In A Bottle consortium^19,21^ (GIAB) because of the integration of multiple variant calling program on a bunch of datasets, and the special process steps on disconcordant variant sites among the programs. It provided all kinds of necessary data for genome analysis, e.g., reference human genome sequence (FASTA file), binary alignment map (BAM file) built from sequenced reads, high confidence regions (BED file), truth genome variants (VCF file), etc. We used three samples, i.e., HG001, HG002 and HG003, of which Oxford Nanopore (ONT) files are available. All these samples were generated by the same chemistry kits, i.e., SQK-LSK109 library prep kits on the FLO-PRO002 flow cell. And GRCh38 version was used as the reference genome sequence. The original sequencing depths of HG001, Hg002, and HG003 are 44.3x, 52.2x, and 51.6x. Low depth data was then generated by down-sampling original BAM file. Furthermore, 157 public Severe acute respiratory syndrome coronavirus 2 (SARS-CoV-2) samples from NCBI website^20^ were used for further validation.

#### Evaluation metrics

The evaluation is performed on all variants by the predicted label and the ground truth label. Recall, precision and F1 score are used as the evaluation metrics. $t_p$, $f_p$, $f_n$ are used to respectively represent true positive, false positive, and false negative samples. The recall rate is calculated by $Recall={t_p}/{(t_p+f_n)}$. The precision is calculated by $Precision={t_p}/{(t_p+f_p)}$. The F1 score is calculated by $F1=2\times (Recall \times Precision)/(Recall+Precision)$. And we used the submodule vcfeval in RTG Tools version 3.9^25^ to generate these three metrics.

#### Hyper-parameters selection

The size of the flanking window is 16. The algorithms are implemented using Pytorch^26^ with an NVIDIA Tesla P100 GPU. We use Adam optimizer^27^ with an initial learning rate of 0.0003. Each mini-batch contains 5000 samples. For each training period, we train the LDV-Caller model up to 30 epochs which takes 36 hours. When inference, analysing whole human genome needs 380 minutes.

#### Training and testing

We first extract all genome sites with minimum of 0.2 allele frequency. These potential sites and truth variant sites are used to extract pileup images. For each site, low depth image *I* and corresponding high depth image $I_{gt}$ are generated. Labels for variant caller and variant discriminator are calculated from truth variant VCF file provided by GIAB consortium. Image *I*, $I_{gt}$ and labels compose the training data. Our LDV-Caller is trained in an end-to-end style. However, parameters of three sub-models in LDV-Caller are supervised by two optimizers. One is for adversarial discriminator. The other optimizes generative model and variant caller at the same time. As suggested in^24^, these two optimizers alternately work in the training process. Moreover, in this work, we give them the same training iterations.

In the testing stage, the adversarial discriminator is not required. At first, we need to extract all potential variant candidates, i.e., all genome sites with minimum of 0.2 allele frequency. Then pileup images are generated from low depth sequencing data at these sites. And 2D low depth images are obtained by reshaping 3D pileup images. The generative model takes these 2D low depth images as inputs and recovers high depth images. Both predicted high depth images and corresponding low depth images are passed to the variant caller. Lastly, based on predicted variant attributes from the variant caller, variants are yielded via using post-processing algorithm in^16^.

### Ethical approval

For the whole experiments, we (1) identify the institutional and/or licensing committee approving the experiments, including any relevant details, (2) confirm that all experiments were performed in accordance with relevant guidelines and regulations.

### Informed consent

Informed consent was obtained from all participants and/or their legal guardians.

## Data Availability

This study did not generate any unique dataset. All samples used are from public dataset.
